# Surgical Strategies for Closure of Complex Posterior Trunk Defects in Myxoid Pleomorphic Liposarcoma: A Case Report and Literature Review

**DOI:** 10.7759/cureus.103018

**Published:** 2026-02-05

**Authors:** Luis Oscar Gonzalez-Alcocer, Mauricio Gutierrez-Alvarez, Kevin Fuentes-Calvo, Irving Fuentes-Calvo, Maria del Carmen Arrieta-Barragan, Mauro Garibaldi Bernot, Cuahutemoc Marquez-Espriella, Jorge Alberto Robles-Aviña

**Affiliations:** 1 General Surgery, Medica Sur, Mexico City, MEX; 2 Plastic and Reconstructive Surgery, Hospital Central Sur Pemex, Mexico City, MEX; 3 Surgery, Medica Sur, Mexico City, MEX; 4 Plastic and Reconstructive Surgery, Hospital Central Sur de Alta Especialidad Pemex, Mexico City, MEX; 5 Surgical Oncology, Medica Sur, Mexico City, MEX

**Keywords:** liposarcoma, pleomorphic myxoid liposarcoma, surgical management, thoracic reconstruction, thorax reconstruction

## Abstract

Myxoid pleomorphic liposarcoma (MPL) is an exceptionally rare subtype of liposarcoma characterized by an aggressive clinical course. Since its first description in 2009, fewer than a dozen cases have been reported, predominantly in pediatric populations and mediastinal locations. Reports in elderly patients and with posterior trunk involvement are exceedingly uncommon.

We report the case of an 83-year-old male with a two-year history of progressive cervical-thoracic swelling, ultimately diagnosed as MPL on biopsy. Imaging revealed an 8.6 × 6.4 × 7.6 cm mass originating from the right cervico-thoracic paravertebral muscles, infiltrating trapezius, rhomboid, subscapularis, and latissimus dorsi. The patient underwent en bloc tumor excision, including paraspinal muscles and partial vertebral elements, followed by immediate reconstruction using regional rotational musculocutaneous flaps (latissimus dorsi and trapezius) performed by a combined oncologic and reconstructive surgical team. Postoperative recovery was uneventful, and the patient was discharged with preserved functional mobility and no wound complications.

MPL is recognized for its high recurrence rate and poor survival. Complete surgical resection remains the cornerstone of treatment, particularly when neoadjuvant therapy is not feasible or ineffective. Reconstruction of large posterior trunk defects is technically demanding due to limited tissue elasticity and the scarcity of recipient vessels for free flaps. Regional pedicled options, including trapezius, latissimus dorsi, and paraspinous flaps, remain reliable strategies. This case emphasizes the feasibility of achieving both oncologic clearance and functional coverage in elderly patients with extensive defects.

This case highlights the rarity of MPL in advanced age and posterior trunk location, underscoring the critical role of individualized reconstructive planning. Regional pedicled flaps provide safe and effective coverage, ensuring functional preservation and reduced morbidity following radical resection.

## Introduction

Myxoid pleomorphic liposarcoma (MPL) is an exceptionally rare subtype of liposarcoma, accounting for fewer than 5% of all liposarcoma variants and predominantly affecting pediatric populations [1]. It is characterized by an aggressive malignant behavior, with reported high rates of local recurrence and metastatic potential. MPL most commonly arises in the mediastinum, a location described in the majority of published cases. Since its recognition as a distinct entity in 2014, fewer than 15 cases have been reported worldwide, underscoring its exceptional rarity.

To the best of our knowledge, this is the first reported case of MPL originating in close contact with the cervical vertebrae in an 83-year-old male. The unusual anatomical location and advanced age of the patient highlight the importance of recognizing reconstructive strategies applicable to atypical clinical scenarios. This report contributes to expanding the limited body of literature on MPL while emphasizing the role of tailored reconstructive approaches in managing complex thoracic and cervical defects within plastic and reconstructive surgery.

## Case presentation

An 83-year-old male patient with no significant medical history presented with a progressively enlarging mass in the cervicodorsal region over a two-year period, which gradually increased in size and resulted in visible deformity at the level of the C7 vertebra.

On physical examination, a soft, poorly mobile mass measuring approximately 20 × 20 cm was observed in the cervicodorsal area, with poorly defined and irregular margins, without evidence of neurological compression or alterations in muscle strength or limb mobility. An incisional biopsy was performed, and histopathological analysis reported a MPL.

A non-contrast computed tomography (CT) scan was subsequently performed, revealing a cystic-appearing nodular lesion apparently originating from the right cervicodorsal paravertebral muscles, teres major, and right trapezius muscle, measuring approximately 8.6 × 6.4 × 7.6 cm.

**Figure 1:**
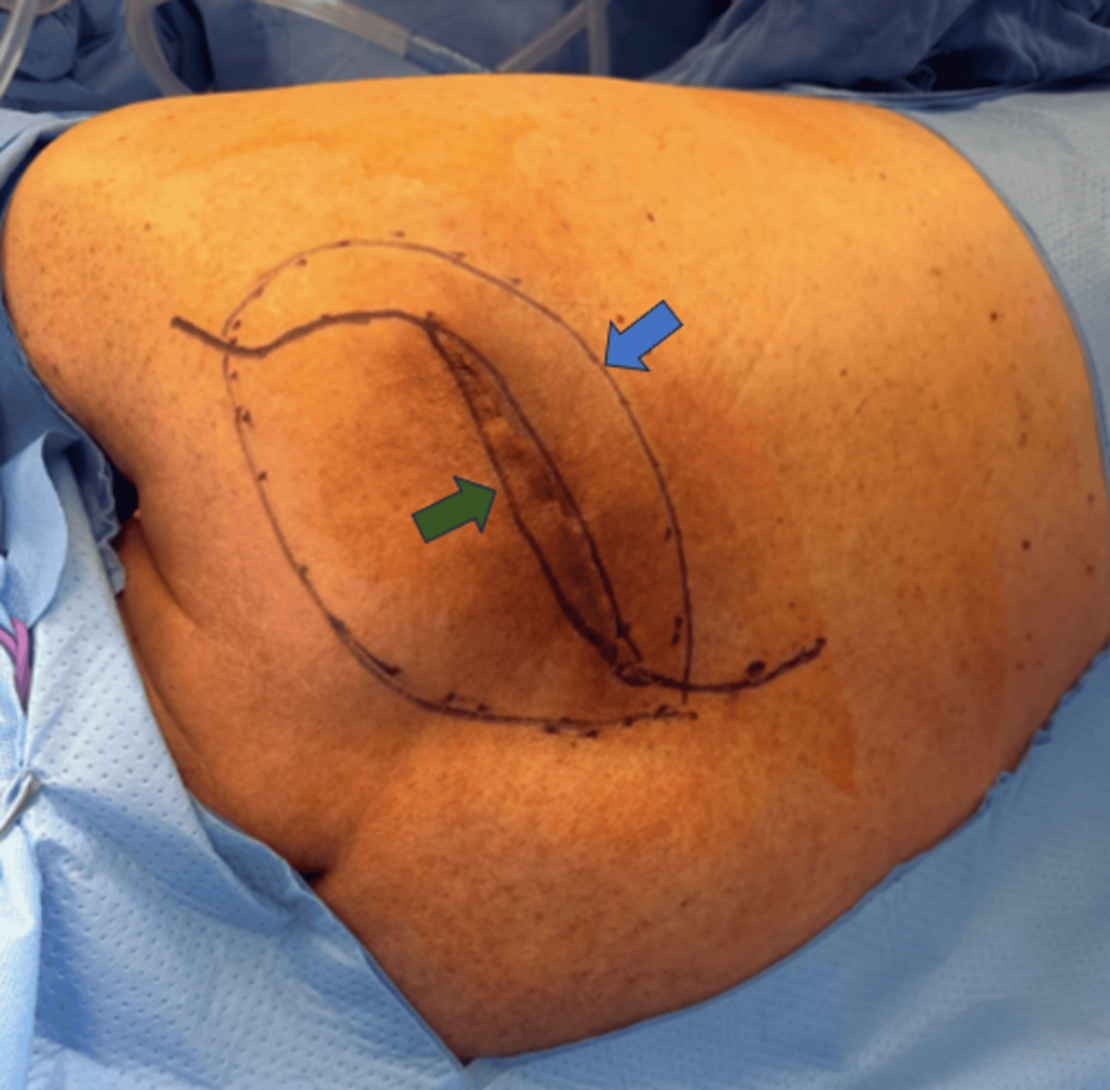
Patient in left lateral decubitus position; the cephalic region is on the left side of the image, and the caudal region on the right The tumor, delineated by the circle (blue arrow), is shown with the planned S-shaped incision designed to reduce tension during closure (green arrow).

**Figure 2:**
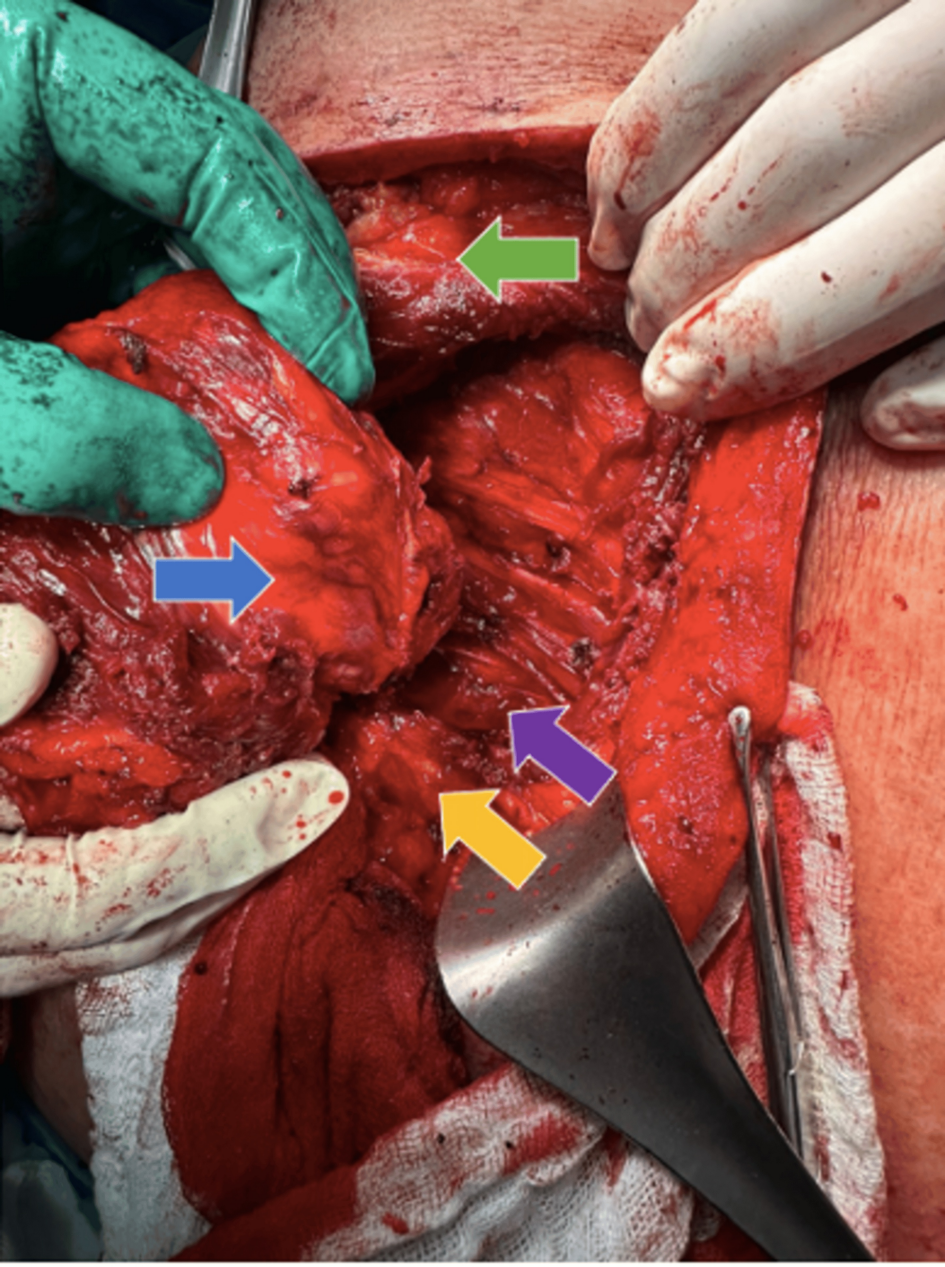
Patient in left lateral decubitus position; the cephalic region is on the left side of the image, and the caudal region on the right The blue arrow indicates the resected tumor, the green arrow the scapula, the yellow arrow the vertebral column, and the purple arrow the paravertebral musculature.

**Figure 3:**
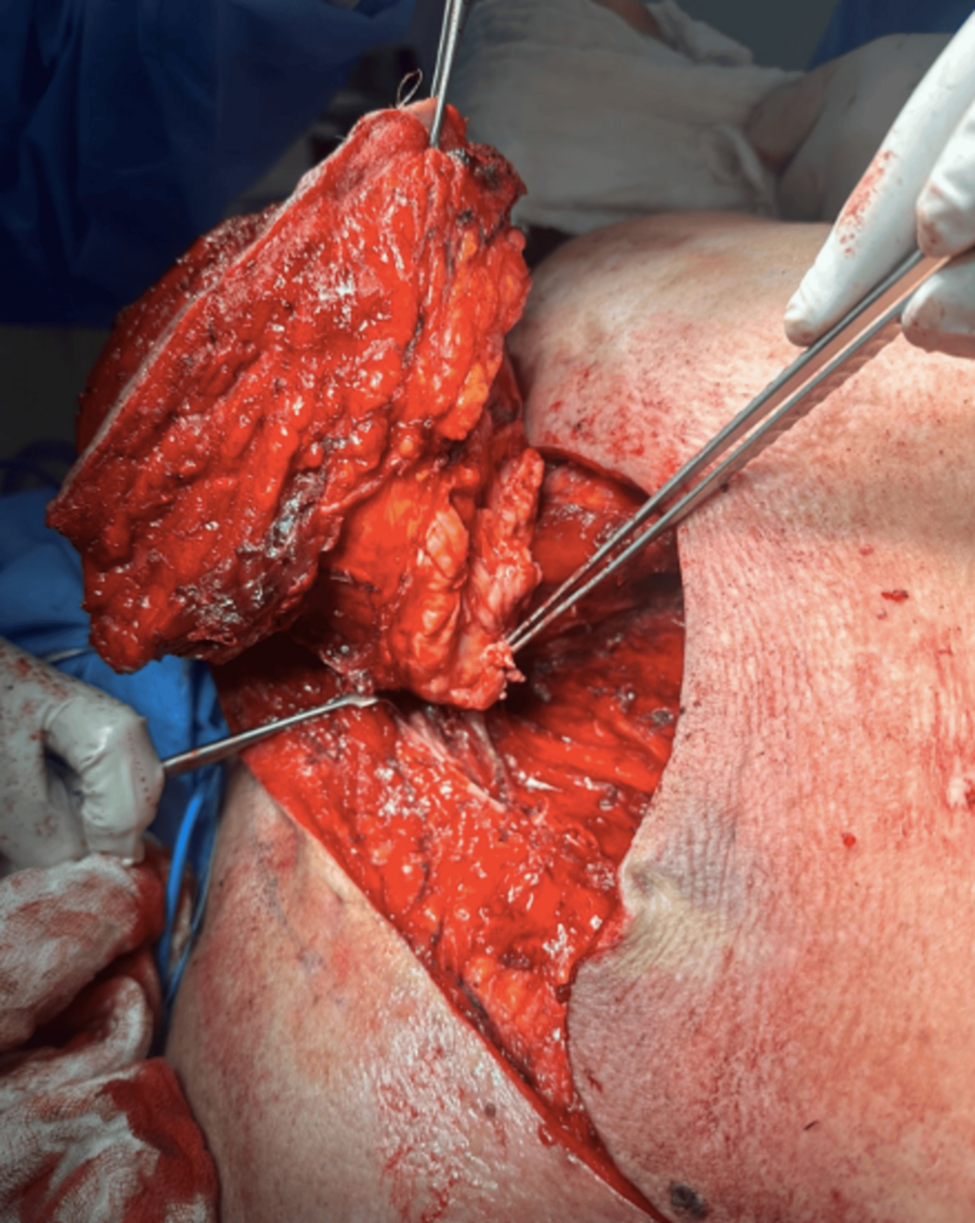
Tumor completely resected

Surgical resection and reconstruction were performed (Figure 1). Intraoperatively, it was necessary to resect the transverse processes of the thoracic vertebrae and perform an en bloc wide excision of the paravertebral, rhomboid, subscapular, and latissimus dorsi muscles, including macroscopically uninvolved surrounding tissue, to achieve oncologic margins. Resection boundaries were determined intraoperatively based on tumor infiltration and adherence to adjacent structures. Dorsal and paravertebral reconstruction was achieved using rotational musculocutaneous flaps, specifically a latissimus dorsi flap combined with paraspinous muscle flaps, providing adequate coverage of the defect and durable soft-tissue support (Figures 2, 3).

**Figure 4:**
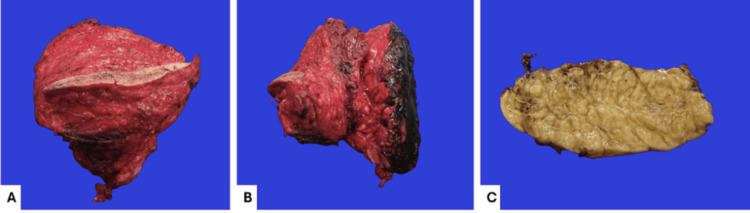
Gross pathology of the specimen Resection of a cervicodorsal tumor measuring 14.0 × 13.5 × 6.5 cm in total (A and B). Cross-sections show a well-defined ovoid lesion with irregular, multinodular borders measuring 13.0 × 7.7 × 6.2 cm and a pale-yellow coloration (C).

The pathology department received a specimen corresponding to a cervicodorsal tumor resection measuring 14.0 × 13.5 × 6.5 cm in total and weighing 581 g. The specimen consisted of an ovoid tumor measuring 13.0 × 7.7 × 6.2 cm, with a rough, yellow, adipose-like surface. At its apical portion, a segment of skin measuring 11.0 × 1.3 cm was attached; it was rough, brownish-white, opaque, and without macroscopic lesions. The surgical bed was inked black. On sectioning, the tumor showed a soft to gelatinous consistency, well-defined margins with a multinodular, pale-yellow, glistening appearance, and gelatinous areas in contact with the surgical bed, located 2.3 cm from the skin surface (Figure 4).

**Figure 5:**
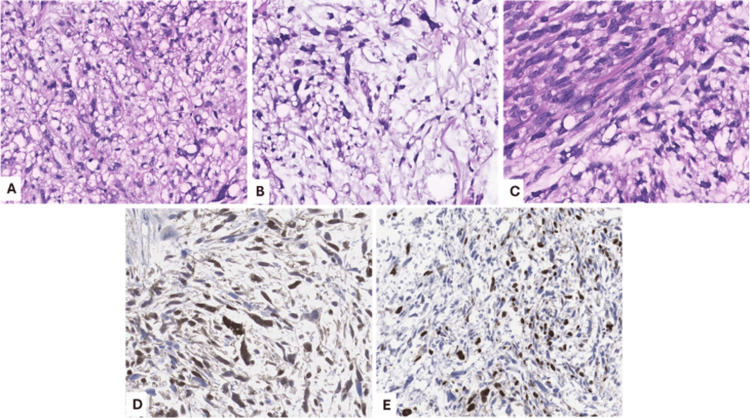
All histological sections with 15× magnification Hematoxylin and eosin staining (A-C) and immunohistochemistry panel (D-E). Pleomorphic lipoblasts and oval cells with hyperchromatic nuclei (A); myxoid areas containing pleomorphic lipoblasts (B); spindle cells with hyperchromatic nuclei within the myxoid matrix component (C); neoplastic cells showing nuclear positivity for S100 (D). The proliferation index, assessed by Ki-67 (MIB1), is 30% (E).

Histologically, pleomorphic vacuolated lipoblasts were identified, accompanied by spindle and oval cells with hyperchromatic nuclei within a myxoid matrix component, along with areas of necrosis and variable mitotic activity. Immunohistochemically, the neoplastic cells showed positivity for p16 and S100, confirming the lipogenic component, and negativity for CDK4, with a Ki-67 proliferation index greater than 30%. The literature describes this tumor subtype as having a nonspecific immunophenotype; however, its morphological features are consistent with the diagnosis (Figure 5).

The postoperative course was uneventful. The patient remained hemodynamically stable, with no evidence of neurological deficit, wound complications, or flap compromise. Adequate pain control and progressive functional recovery were achieved, allowing safe discharge in stable condition due to clinical improvement on the fifth day.

## Discussion

Liposarcoma is a malignant neoplasm of adipocytic origin, historically divided by the World Health Organization (WHO) into four principal subtypes: well-differentiated, dedifferentiated, pleomorphic, and myxoid [1]. In 2009, Alaggio et al. described MPL as a distinct entity, characterized by histological features that overlap those of conventional myxoid liposarcoma and pleomorphic liposarcoma [2,3]. This subtype is exceedingly rare; since 2014, only 10 cases have been reported worldwide [4-13], underscoring its exceptional rarity (Table 1), with most reported cases occurring in infants and young adults, predominantly affecting females, in contrast to our case, which involves an 83-year-old male patient. The mediastinum has been recognized as the most common primary site, although other anatomical regions may be involved [14], as in our case, with an atypical presentation of the tumor in the cervicodorsal region.

**Table 1:** Reported cases of MPLPS in the literature, 2014-2025 This table summarizes all individual case reports of myxoid pleomorphic liposarcoma (MPLPS) published between 2014 and 2025. Data were extracted from PubMed/MEDLINE, Scopus, and Google Scholar up to August 2025 using the search terms “myxoid pleomorphic liposarcoma” AND “case report.” Only single-patient case reports providing clinical, pathological, genetic, therapeutic, and/or outcome information were included. Series and reviews were excluded from the tabulation but were consulted for context. IHC, immunohistochemistry; not reported, information not specified in the article

No	First author	Year	Title	Journal	Country	Age (years)	Sex	Location	Size (cm)	Genetics/IHC	Treatment	Outcome
1	Ahmad et al. [4]	2025	Myxoid pleomorphic liposarcoma of the posterior chest wall: a case report	Radiol Case Rep	Pakistan	35	M	Posterior chest wall	Not reported	Not reported; typically TP53/RB1 altered, MDM2/CDK4 negative	Surgical resection	Not reported
2	Tseng et al. [5]	2024	Myxoid pleomorphic liposarcoma presenting as diffuse intra-abdominal sarcomatosis in a patient with Li-Fraumeni syndrome	BMJ Case Rep	Taiwan	20	F	Intra-abdominal (diffuse)	Not reported	Germline TP53 mutation (Li-Fraumeni)	Palliative cytoreduction	Not reported
3	Chandrasekaran et al. [6]	2024	Myxoid pleomorphic liposarcoma of the spermatic cord: a rare case report	Cureus	India	50	M	Spermatic cord (paratesticular)	4 × 4 × 3.5	CD34+, p53+; focal S100; MDM2/CDK4 negative	High inguinal orchiectomy + doxorubicin chemotherapy	Disease-free at 2 months
4	Tan et al. [7]	2024	Myxoid pleomorphic liposarcoma of the orbit: a case report and review	Pathology	Australia	12	F	Orbit	Not reported	p53 mutated; DDIT3−; MDM2/CDK4−	Excision + chemotherapy	No metastasis at diagnosis
5	Shen et al. [8]	2024	Myxoid pleomorphic liposarcoma: a report of a case with novel fusion genes and review of the literature	J Clin Pathol	China	Not reported	F	Abdomen	Not reported	TP53 and RB1 mutated; CREB5::TERT and ETV1::LFNG fusions; MDM2/CDK4 negative	Complete resection, no radio/chemotherapy	Recurrence-free at 18 months
6	AlObaid et al. [9]	2022	Myxoid pleomorphic liposarcoma of the falciform ligament: a case report	J Surg Case Rep	Saudi Arabia	58	F	Falciform ligament	Not reported	Not reported	Surgical resection	Not reported
7	Gami et al. [10]	2021	Myxoid pleomorphic liposarcoma in an infant: a case report	Int J Surg Case Rep	Nepal	1	M	Hemithorax/left chest wall	10.5 × 9.7 × 6.6	S100+, CD34+; MDM2/CDK4 negative	Thoracotomy + doxorubicin/cyclophosphamide chemotherapy	Alive and asymptomatic at 5 months
8	Zare et al. [11]	2020	Myxoid pleomorphic liposarcoma in a patient with Li-Fraumeni syndrome	Int J Surg Pathol	Iran	34	M	Anterior chest wall	2.9 × 2.3 × 2.0	Germline TP53 mutation (Li-Fraumeni); DDIT3−; MDM2/CDK4−	Not reported	Not reported
9	Sinclair et al. [12]	2017	Myxoid pleomorphic liposarcoma of the perineum in a 15-year-old girl with Li-Fraumeni syndrome	Pediatr Surg Int	USA	15	F	Perineum	Not reported	Germline TP53 mutation (Li-Fraumeni)	Not reported	Not reported
10	Creytens et al. [13]	2014	Myxoid pleomorphic liposarcoma: evidence for its entity using array comparative genomic hybridization	J Clin Pathol	Belgium	Not reported	Not reported	Not reported	Not reported	Arr ay-CGH; multiple chromosomal alterations	Not reported	Not reported

MPL is clinically relevant due to its aggressive biological behavior, with high rates of local recurrence and poor overall survival [15]. At the six-month follow-up, our patient has shown no clinical or radiological evidence of recurrence, suggesting that a wide and complete resection of the tumor may be effective in reducing local recurrence rates. Given its rarity, knowledge regarding optimal management strategies remains limited. Therefore, a multidisciplinary management of this entity is necessary, involving oncologic surgery, medical oncology, and plastic surgery for defect reconstruction, particularly in cases of large tumors such as the one presented here.

Neoadjuvant therapy is typically indicated, particularly for those of high grade, exhibiting more than 25% dedifferentiated morphology or greater than 5% round cell morphology on histology. Clinical studies suggest that under these conditions, neoadjuvant treatment may improve outcomes, with reported five-year survival rates of approximately 64% [15]. Nevertheless, in cases where the tumor demonstrates resistance or inadequate response to chemotherapy, complete surgical resection with negative margins also remains the mainstay of therapy in the curative treatment.

Multidisciplinary management is essential because soft-tissue defects of the posterior trunk may represent challenges for reconstruction. Reasons include shortages of both reliable axial pattern flaps for local tissue transfer and recipient vessels for microsurgical reconstruction. Given the relative lack of elasticity as well as a shortage of potential microsurgical recipient vessels, the back offers special challenges to the surgeon [16], which makes the involvement of a plastic surgeon necessary.

Nevertheless, the dorsal trunk hosts several muscles that may be transferred as pedicled flaps, such as M. latissimus dorsi or M. trapezius flaps. A popular fasciocutaneous option is the parascapular flap; though given their cephalic pedicle, they are not useful for reconstruction of the lower back. Moreover, in selected cases, free flaps with vein grafts or loops may be utilized. More recent trends involve the application of fasciocutaneous perforator flaps since the posterior trunk involves abundant perforators [17].

**Figure 6:**
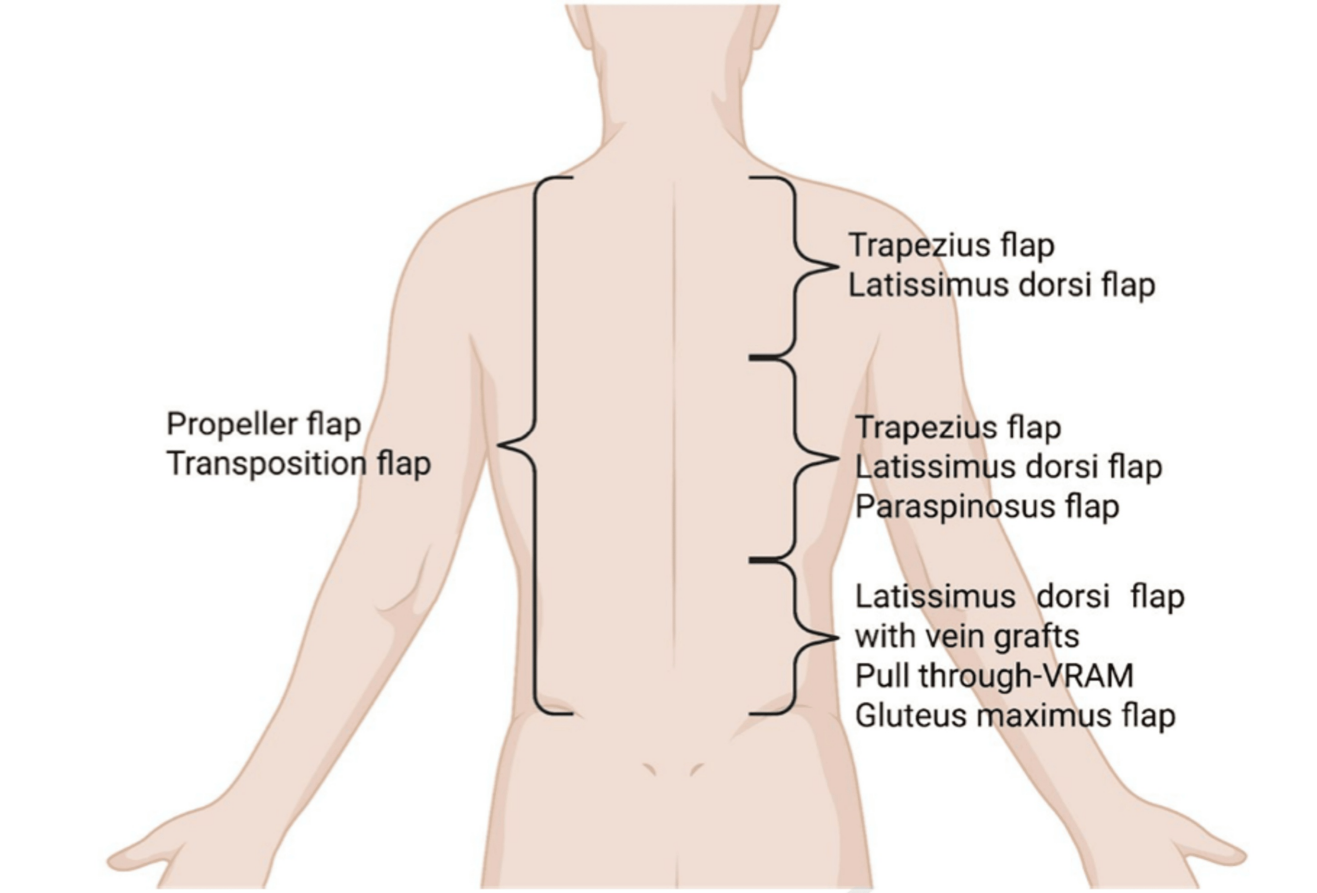
Regional flap options for posterior trunk and chest wall reconstruction Illustration summarizing the main regional flaps available for posterior trunk and thoracic wall reconstruction following extensive oncologic resection. Options vary according to the anatomical level of the defect: superior (trapezius and latissimus dorsi flaps), middle (trapezius, latissimus dorsi, and paraspinosus flaps), and inferior (latissimus dorsi flap with vein grafts, pull-through VRAM, and gluteus maximus flap). Additional coverage may be achieved with propeller and transposition flaps, depending on defect extension and tissue availability. VRAM, vertical rectus abdominis myocutaneous Image credit: authors of this article.

Figure 6 illustrates the transposition pattern and the perforator flaps.

The posterior trunk, due to its extensive surface area and rich network of perforating vessels, provides a wide spectrum of reconstructive possibilities utilizing random pattern and, more importantly, perforator-based flaps. When fundamental surgical principles are observed, such as maintaining an adequate width-to-length ratio for random-pattern flaps and respecting the appropriate angiosome dimensions in perforator flaps, these techniques offer reliable coverage and can effectively address a broad range of posterior trunk defects.

Among the pedicled fasciocutaneous flaps available for posterior trunk reconstruction, the parascapular and scapular flaps are notable options. Both are vascularized by the circumflex scapular artery and are suitable for coverage of defects in the upper and mid-thoracic regions. Similar to free flap procedures, the vascular pedicle can be dissected proximally to the subscapular artery and vein. Parascapular flaps can reach dimensions of up to 15 × 40 cm, providing substantial coverage. Additionally, gluteal perforator flaps are valuable for reconstructing sacral defects, with the superior gluteal artery perforator (S-GAP) flap offering reliable tissue transfer while preserving the integrity of the gluteus maximus muscle [16].

Supraclavicular flaps

The supraclavicular flap, which may also be harvested as an island flap, constitutes a versatile fasciocutaneous option for soft-tissue reconstruction of the posterior cervical region. As its name suggests, it is designed from the supraclavicular area and is vascularized by the transverse cervical artery. These flaps can be elevated with dimensions reaching up to 22 cm in length and 10 cm in width, providing reliable coverage for defects in this anatomical zone [16,18].

Trapezius flap

The trapezius muscle, located superficially in the neck and upper thoracic region, represents a valuable option for reconstruction of defects in the upper thorax and cervical area. Its vascular supply is divided regionally: the inferior portion is perfused by the dorsal scapular artery, a deep branch of the cervical system, while the transverse segment receives blood from the superficial cervical artery. Anatomically, the muscle extends from the external occipital protuberance to the level of the 12th thoracic vertebra, with potential dimensions of up to 34 × 18 cm. It may be harvested either as a muscle-only flap or as a myocutaneous flap. Dissection can be performed in a caudal-to-cephalic direction, allowing for rotation into defects of the upper posterior trunk and dorsal neck. Furthermore, the trapezius flap may be employed as an advancement flap or as a turnover flap, offering considerable versatility in reconstruction [16,19].

Latissimus dorsi flap

The latissimus dorsi flap provides a versatile and reliable option for reconstructing extensive defects of the mid- and upper-thoracic posterior trunk [16,19]. It can be harvested with dimensions of up to 30 × 40 cm and may be utilized either as a muscle flap, often combined with skin grafts, or as a myocutaneous flap. Anatomically, the latissimus dorsi originates from the thoracic spinous processes, lower ribs, and iliac crest, and inserts into the intertubercular groove of the humerus. Its principal vascular supply arises from the thoracodorsal artery, a branch of the scapular vascular system, while secondary contributions are provided by intercostal and lumbar arteries. This flap is particularly advantageous as it can be rotated to cover contralateral or more caudal defects, offering broad applicability in posterior trunk reconstruction [19].

Gluteus maximus flap

In addition to gluteal perforator flaps, sacral region defects can also be effectively reconstructed using the gluteus maximus muscle flap. The superior gluteal artery serves as a reliable vascular pedicle, supporting both advancement and turnover configurations of the gluteus maximus flap for coverage of sacral defects [20].

Microsurgery and recipient vessel options

In exceptional cases where posterior trunk defects cannot be adequately managed with local or pedicled flaps, microvascular free flaps become necessary. While donor site availability is virtually unlimited, identifying suitable recipient vessels often poses a greater challenge. The superior gluteal artery in the buttock region represents one possible recipient vessel. Alternatively, free flaps may be anastomosed to the fourth lumbar artery, located lateral to the sacrospinal muscle. When these options are not feasible, vein graft interposition may be required. Few et al. described their experience in managing the so-called “hostile back,” characterized by defects exceeding 200 cm², prior irradiation, severe infection, or exposed hardware [21]. In their series, reconstruction was achieved using free latissimus dorsi or vertical rectus abdominis myocutaneous (VRAM) flaps in combination with interposed vein grafts, with the great saphenous vein serving as a reliable conduit.

## Conclusions

MPL is an extremely rare soft-tissue sarcoma with overlapping histologic features of myxoid and pleomorphic liposarcoma. Its management poses a unique reconstructive challenge, particularly when located in the cervicodorsal region, as in our case. Complete oncologic resection with adequate margins remains the mainstay of treatment and may contribute to lower recurrence rates.

From a reconstructive standpoint, extensive defects of the posterior trunk demand individualized planning by the plastic surgeon. The use of local and regional flaps, such as the latissimus dorsi, trapezius, or parascapular flaps, offers reliable coverage and preservation of function, minimizing donor-site morbidity. This case highlights the essential role of plastic surgery in achieving both oncologic safety and durable reconstruction, emphasizing the importance of multidisciplinary collaboration in the management of rare and complex tumors such as MPL.
